# Glycaemic control and novel technology management strategies in pregestational diabetes mellitus

**DOI:** 10.3389/fendo.2022.1109825

**Published:** 2023-01-12

**Authors:** Christine Newman, Adesuwa Ero, Fidelma P. Dunne

**Affiliations:** ^1^ School of Medicine, College of Medicine, Nursing and Health Science, University of Galway, Galway, Ireland; ^2^ Department of Diabetes and Endocrinology, Galway University Hospital, Galway, Ireland; ^3^ Diabetes Collaborative Clinical Trials Network, University of Galway, Galway, Ireland

**Keywords:** pregnancy, technology, continuous glucose monitoring (CGM), continuous subcutaneous insulin infusion (CSII), pumps

## Abstract

**Introduction:**

Pregestational diabetes (PGDM) is an increasingly common and complex condition that infers risk to both mother and infant. To prevent serious morbidity, strict glycaemic control is essential. The aim of this review is to review the glucose sensing and insulin delivering technologies currently available for women with PGDM.

**Methods:**

We reviewed online databases for articles relating to technology use in pregnancy using a combination of keywords and MeSH headings. Relevant articles are included below.

**Results:**

A number of technological advancements have improved care and outcomes for women with PGDM. Real time continuous glucose monitoring (rtCGM) offers clear advantages in terms of infants size and neonatal intensive care unit admissions; and further benefits are seen when combined with continuous subcutaneous insulin delivery (insulin pump) and algorithms which continuously adjust insulin levels to glucose targets (hybrid closed loop). Other advancements including flash or intermittent scanning CGM (isCGM) and stand-alone insulin pumps do not confer as many advantages for women and their infants, however they are increasingly used outside of pregnancy and many women enter pregnancy already using these devices.

**Discussion:**

This article offers a discussion of the most commonly used technologies in pregnancy and evaluates their current and future roles.

## 1 Introduction

Pregestational diabetes mellitus (PGDM) is a combination of complex, chronic conditions defined by hyperglycaemia and associated with adverse fetal and maternal complications. PGDM includes any form of diabetes which exists before the conception. The most common forms of PGDM are undoubtedly type 1 and type 2 diabetes, however other forms of diabetes including latent autoimmunity diabetes of adulthood (LADA), maturity onset diabetes of the young (MODY), cystic fibrosis related diabetes (CFRD) and diabetes related to endocrinopathies and medications can occur in women of childbearing age and can cause complications during pregnancy.

Type 1 and type 2 diabetes complicate between 0.5-2.4% of all pregnancies worldwide (1). While there is variation in prevalence across different regions, there has been a universal increase in the number of pregnancies complicated by PGDM and its rate has more doubled since 1995 (1, 2). This substantial increase is likely multifactorial. Firstly more women are entering pregnancy with type 2 diabetes and women with type 2 diabetes now account for between 30-50% of cases of PGDM (3, 4). Rates of type 2 diabetes have increased by roughly 30% in recent years (5) – most likely due to the increase in obesity in adolescents and young adults (6). Other factors contributing to the rise of type 2 diabetes include urbanisation, environmental factors like pollution and increased testing and detection (7). The incidence of type 1 diabetes, which is similarly rising by roughly 1.9% per year is less well understood (8).

The prompt recognition and treatment of PGDM is important due to both the short and long term complications faced by both mother and infant. During pregnancy, complications such as pre-eclampsia and Caesarean delivery are three times more common than the non-diabetic population (9) and half of women with PGDM will have at least one hospitalisation during their pregnancy (3). Infants are at risk of preterm delivery (OR 3.48); macrosomia (OR 1.51); being born large for gestational age (LGA) (OR 3.9) and have a 2-3.5 fold increased risk of neonatal death and stillbirth (10). Much of this risk of perinatal mortality comes from the increased risk of congenital anomalies seen in the infants of diabetic mothers (9). In the long term, infants of women with diabetes are more likely to be overweight and obese in childhood and display evidence of significant insulin resistance (even when adjusted for confounders like family history) (11). Similarly rates of cardiovascular disease are 29% higher in infants exposed to PGDM and these infants have a higher rate of hypertensive disorders and venous thromboembolism (10). The risk of complications in the offspring also seems to be associated with the number of diabetes related complications in the mother (12). More recently the risk of autism, attention-deficit hyperactivity disorder (ADHD) and other neurocognitive disorders have been increasingly recognised. A recent meta-analysis identified hazard ratios of 1.36 (95% CI 1.19-1.55) and 1.98 (95% CI 1.46-2.88) for ADHD and autism respectively (13). This risk appears to correlate with the degree of fetal exposure to hyperglycaemia as neurocognitive disorders are more common in type 1diabetes, and it is thought to be directly related to the effect of hyperglycaemia on the developing brain and neural pathways (14). Similarly insulin use in GDM is associated with neonatal hypoglycaemia and worse neonatal neural adaptability (15).

The serious and diverse range of complications faced by this cohort make the long term follow up of infants exposed to diabetes all the more important (16).

Strict glycaemic control is key to the prevention of many of these complications and the cornerstone of management. International guidelines like the American Diabetes Association (ADA) and the National Institute for Health & Care Excellence (NICE) recommend that women with PGDM who are planning a pregnancy aim for a HbA1c level below 48 mmol/mol (6.5%), if attainable without causing significant hypoglycaemia, to reduce the risk of complications associated with elevated glucose (17, 18). In pregnancy, the ADA recommends a HbA1c target of < 42mmol/mol (<6%) but highlights that this target can be relaxed to < 53mmol/mol (<7%) to prevent hypoglycaemia. These targets were selected as HbA1c levels above this level have been shown to be associated with an increase in adverse outcomes. A peri-conception HbA1c above 49mmol/mol (6.6%) [adjusted odds ratio, aOR=1.02 (95% CI: 1.00 - 1.04)], pre-pregnancy retinopathy [aOR=2.05 (95% CI: 1.04 - 4.05)] and lack of pre-pregnancy folic acid consumption [aOR=2.52 (95% CI: 1.12 - 5.65)] were all independently associated with increased odds of fetal and infant death (19). Observational population based studies have also demonstrated the increased risk of congenital anomaly that correlates directly with poor glycaemic control (20). Interventions including pre-pregnancy care (PPC) have successfully improved maternal and fetal outcomes and are cost-effective (21). A systematic review and meta-analysis of observational studies evaluating the effectiveness of PPC in improving maternal and perinatal outcomes suggested that PPC is associated with a reduction in first trimester HbA1c of 1.27% (22)- it is however worth noting that this was in high income countries with homogenous populations. The meta-analysis results showed that attendance at PPC reduced congenital malformation risk by 71%, [RR=0.29 (95% CI: 0.21–0.40)]. It also resulted in a reduction in the risk of preterm delivery by 15%, [RR=0.85 (95% CI: 0.73–0.99)] and a risk reduction of perinatal mortality by 54%, [RR=0.46 (95% CI: 0.30–0.73)]. Results of these and other studies which have demonstrated the importance of good pre- and ante-natal glycaemic control highlighted the need for better treatment options and changes in care for women with PGDM.

As for non-pregnant adults, advances in glucose sensing and insulin delivery technology offer potential improvements in care. From the use of rudimentary and cumbersome insulin pumps in the 1960 (23–27), to sophisticated, advanced hybrid closed loop insulin delivery systems (28), the treatment of diabetes has dramatically changed since the discovery of insulin in 1921 (29). A summary and timeline of the evolution of diabetes treatment for non-pregnant adults can be found in **Table 1**.

**Table 1:** Advances in diabetes technology for non-pregnant adults with type 1 diabetes mellitus.

Year	Advancement	Improvements
1921 (29)	Discovery of insulin by Banting and Best	Provided treatment for a previously fatal condition
1920-1930 (30)	Protamine and zinc were successfully added to insulin to increase its duration of action	Fewer daily injections required for patients
1963 (31)	Dextrostix were discovered and were available to patients	Paper strip allowing as assessment of glucose concentration against a graded colour chart
1970 (31)	Introduction of Ames Reflectance Meter	Allowed rapid assessment of blood glucose -for physician use only
1970-1980 (23–27, 30, 32, 33)	Developments made human insulin commercially available, rudimentary insulin pumps became available, pre-filled insulin pens became available, and advancements in glucometers meant patients were able to monitor glucose at home	Commercially available pumps were modelled off research prototypes and were heavy, expensive and packs required frequent charging thus making them largely impractical
1993 (34)	DCCT trial was published	The importance of strict glycaemic control was definitively demonstrated in type 1 diabetes
1999-2008 (35)	First, second and 3^rd^ CGM systems became available	Further choice for patients, required calibration every 10 hours
2009 (36)	Insulin pumps with threshold suspend features were introduced	Safety net for people with hypoglycaemia which is superior to sensor augmented pump alone (2013)
2010 (37)	Sensor augmented pumps were shown to be superior to multiple daily injections in terms of reducing HbA1c	Insulin pumps with predictive alarms helped prevent hypo and hyperglycaemia
2012 (38)	CGM added to CSII showed benefit over SMBG	Added strength to the argument that CGM showed be made more widely available
2014 (39)	Intermittently scanned CGM becomes available (isCGM)	Improved hypoglycaemia and satisfaction in adults with type 1 diabetes
2014 (40)	Open APS system was launched	People with type 1 diabetes could access algorithms to create their own hybrid closed loop or artificial pancreas system (also known as DIYAPS or open source automated insulin delivery)
2017 (41)	Hybrid closed loop technology becomes available	Closed loop demonstrated improved glycaemic control and allowed greater choice for patients
2018	First Food and Drug Administration (FDA) approval for implantable CGM	CGM which can remain *in situ* for between 90-180 days.
2019 (42)	Further hybrid closed loop technology becomes available	Closed loop technology showed superiority against sensor augmented pump
2022 (43–45)	1. Four commercially available closed loop systems available in the UK 2. “Bionic pancreas” studied in short term studies 3. Open source automated insulin delivery was compared to commercially available sensor augmented pump 4. Intermittently scanned CGM with alarms compared to SMBG	1.Greater potential for patient choice and selection 2. System which delivers automated insulin delivery with meal announcements (but no carb counting) was superior to CGM and any method of insulin delivery over a 13 week period 3. open-source AID system resulted in a significantly higher percentage of time in the target glucose range than the use of a sensor-augmented insulin pump at 24 weeks 4. isCGM with optional alarms for high and low blood glucose levels resulted in significantly lower glycated haemoglobin levels compared to SMBG

### 1.1 Aims

The aim of this article is to summarise the advancements made in the area of diabetes in pregnancy and to highlight the corresponding improvements seen in pregnancy outcomes.

## 2 Technologies

### 2.1 Glucose sensing technology

#### 2.1.1 Real time continuous glucose monitoring

Real time continuous glucose monitoring (rtCGM) offers hundreds of real-time interstitial glucose readings per day and allows the wearer to enable alerts for hypo- and hyperglycaemia. This allows the patient to observe daily patterns in glucose levels to improve long term glycaemic control and to make treatment decisions to avoid hypo- and hyperglycaemia. Through this mechanism rtCGM has been shown to be effective in reducing hypoglycaemia and improving glycaemic control and quality of life for patients with type 1 diabetes (46). In certain countries rtCGM is now commonly used before and throughout pregnancy and has been studied during labour and delivery (47).

A summary of the commonly used terminology in rtCGM (specific to pregnancy) is shown in **Box 1.**

Box 1 **Commonly used terms in diabetes technology (48).**

Time in range (TIR): number of minutes/hours per day spent between 3.5 and 7.8 mmol/L. Patients with diabetes in pregnancy should aim to spend >70% (>16 hours, 48 mins) per day in this range. For non-pregnant adults this range is 4-10 mmol/L.

Time below range (TBR): number of minutes per day spent <3.5 mmol/L. Patients with diabetes in pregnancy should aim to spend <4% (<1 hour) per day in this range; and should aim to spend <1% (<15 mins) per day <3mmol/L.

Time above range (TAR): number of minutes per day spent >7.8 mmol/L. Patients with diabetes in pregnancy should aim to spend <25% (<6 hours) per day in this range.

Although rtCGM has been studied in diabetes since the 1970s and studied in diabetes in pregnancy since at least 2008 (49), the seminal trial in this field was published in 2017. The Continuous glucose monitoring in pregnant women with type 1 diabetes (CONCEPTT) study was a landmark randomized controlled trial in diabetes in pregnancy (50). In this study, 325 women with type 1 diabetes who were pregnant or planning pregnancy were randomized to either rtCGM or self-monitoring of blood glucose (SMBG). Although no improvement was seen in hypoglycemia, a reduction in hyperglycemia in the rtCGM group resulted in a greater number of hours spent in range between 3.5-7.8 mmol/L (TIR). This resulted in improved fetal outcomes including a reduction in LGA births (odds ratio 0·51, 95% CI 0·28 to 0·90; p=0·0210), a reduction in neonatal hypoglycemia (odds ratio 0·45; 0·22 to 0·89) and a reduction in time spent in neonatal intensive care units (NICU). Further *post-hoc* analyses of the CONCEPTT study demonstrated:

***a)*** even slight increases in the number of minutes per day spent in the target range in trimesters two and three can decrease the risk of neonatal hypoglycemia (51)
***b)*** hyperglycemia in the early morning and late afternoon in trimester two and three respectively can increase rate of LGA (52) – underlining the importance of analyzing patterns and adjusting insulin regimes and
***c)*** Post-prandial rises are more pronounced in those with LGA infants, again demonstrating the importance of appropriate and timely insulin bolus adjustments (53).

Similar observational studies have demonstrated that each additional 8.5% TIR in pregnancy correlates to a 1% or an 11 mmol/mol decrease in HbA1c (54).

These improvements in fetal outcomes prompted assessments of potential cost benefits and cost-analyses which demonstrated favorable results. The degree of cost-effectiveness differs internationally as the cost associated with NICU varies (55). For example in one Canadian study rtCGM was cost effective when paid for by the individual patient, and cost neutral when paid for by the healthcare provider or government (in this instance, the patient pays for the initial cost outlay and the government does not have to incorporate the cost of purchasing the rtCGM into their budget). In UK based cost-analysis, rtCGM was calculated to be very cost efficient (56). It is important to note that this study included the use of real time CGM rather than retrospective CGM. Retrospective CGM only allows retrospective evaluation and review of glucose readings and does not allow the patient to make changes to their insulin dose at the time of hyperglycemia. Retrospective CGM is very helpful in evaluating daily trends in hyperglycemia, however it has shown conflicting results in terms of fetal macrosomia (57).

In women with type 2 diabetes, studies of rtCGM have been small and findings are inconsistent. One study which enrolled 46 women with type 1 and 25 women with type 2 diabetes found an improvement in birth weight and macrosomia in the rtCGM group, although an exact breakdown was not given (49). A larger Danish study which enrolled 123 women with type 1 and 31 women with type 2 diabetes found no difference between rtCGM and SMBG users (58).

From a patient perspective, rtCGM appears to be generally well tolerated. The main drawbacks of rtCGM include sleep disturbance, discomfort/adhesive issues at the insertion site and false readings of hypoglycemia related to positioning (compression hypoglycemia) (59). Despite this more than 80% would recommend rtCGM and a similar proportion would use it again (60). This increased use and popularity of rtCGM also carries implications for healthcare providers (61). The analysis of rtCGM is resource intense and requires dedicated time with each patient. Patients derive the most benefit from rtCGM when it is reviewed by a clinician and adjustments can be made (62). As such the effective use of rtCGM relies on appropriately trained staff and regular patient contact.

Lastly, although rtCGM unequivocally confers benefits to women with type 1 diabetes, due to its cost it is not universally available and the inequity of available healthcare remains a major issue for people living with diabetes worldwide (63).

#### 2.1.2 Intermittent scanning CGM

Intermittently scanned CGM (isCGM) or flash CGM provides immediate information about a patient’s current and predicted interstitial glucose. It differs from rtCTG as patients have to scan a wearable device with a device reader or use an app on their mobile phone (whereas rtCGM provides constant information without scanning) (64). Unlike rtCGM, first generation isCGMs do not have alarms and do not need to be calibrated against SMBG.

The glucose targets for isCGM are the same as for rtCGM and isCGM devices have also been studied and approved for use in pregnancy.

One measurement tool which is being increasingly used in discussions about real time and intermittent scanning CGM is the mean absolute relative difference (MARD). MARD is a single number which represents the accuracy of the glucose monitor. It is calculated using the difference between the CGM readings and the values measured at the same time by the reference measurement system eg central laboratory level (65).

When compared to SMBG in pregnancy, the MARD between isCGM and SMBG is 11%, and this same study found high rates of patient satisfaction (66). It is worth noting that this was a single study of 74 pregnancy women, however the MARD range is similar to that quoted by the manufactures and real life data (67, 68).

isCGM also has a number of benefits over SMBG including less hypoglycaemia and greater TIR (69, 70). Despite these advantages, discrepancies exist between isCGM and SMBG. In one study isCGM under-estimated glucose levels and potentially lead to differences in treatment decisions in up to 30% of instances (71). Finally improved fetal outcomes have not been demonstrated when compared to SMBG (69). In larger observational studies of over 300 women with type 1 diabetes, isCGM resulted in better glycaemic control in trimester 2 compared to conventional SMBG - however this translated to higher rates of neonatal hypoglycaemia and no improvement in rates of LGA or prematurity (72). The authors have suggested that the tendency of isCGM to under-read glucose readings could make patients more likely to overtreat hypoglycaemia, and thus expose themselves to rebound hyperglycaemia. It may also lead both patients and clinicians to be more cautious about insulin adjustments.

When compared to rtCGM, isCGM has some disadvantages. In one study of 20 pregnant women with type 1 diabetes, isCGM users reported a great TBR overnight and similar results have been found in more recent studies (73, 74). Although there was no significant difference in maternal or fetal outcomes such studies raise the question of using isCGM for treatment decisions, especially overnight. Other studies which evaluated clinical outcomes using older rtCGM and isCGM found better glycaemic control in the first trimester in rtCGM users. Despite better control in this critical period of organogenesis, the clinical outcomes were comparable and patients in both groups improved their glycaemic control (75).

The culmination of this evidence has resulted in the recommendation to offer isCGM to all pregnant women with PGDM who are unable or unwilling to use rtCGM (76).

### 2.2 Insulin delivery technology

#### 2.2.1 Continuous subcutaneous insulin infusion

Despite the clear benefits of CSII outside of pregnancy, in pregnancies complicated by type 1 diabetes the use of CSII has been associated with some disadvantages and conflicting evidence. While initial studies showed benefit in neonatal hypoglycaemia, caesarean delivery, preterm delivery and the Apgar score at five minutes (77), other results have been disappointing. Some studies have failed to demonstrate glycaemic benefits and have found that CSII users require more support and staff resources (78). Others have shown better glycaemic control and less hypoglycaemia, but this has not translated to improved fetal outcomes (79–82). One meta-analysis of four randomised and 43 observational studies found that although glycaemic control and insulin requirements were better in CSII versus multiple daily injections (MDI), rates of gestational weight gain, large and small for gestational age (LGA, SGA) and second and third trimester glycaemic control were inferior in CSII users (83). An older systematic review of RCTs found no differences between CSII and MDI (84). Other studies similarly found higher rates of gestational weight gain and LGA in CSII users despite better glycaemic control (85).

A pre-specified analysis of the CONCEPTT study found that MDI users had better glycaemic control at 24 and 34 weeks, had less hypertension and less neonatal hypoglycaemia and NICU admissions (86). A similar study which also evaluated CSII and MDI in rtCGM users found no significant difference in outcomes (87).

It is worth noting that much of this evidence comes from observational studies and the lack of RCTs in this area mean that results must be interpreted in this light.

CSII have also been studied at the time of delivery and women who consistently use CSII throughout pregnancy and delivery have better TIR than those who switch to an intravenous insulin infusion in labour. It should be noted however that the decision to use CSII during labour is multifactorial and depends on staff familiarity with different diabetes technology and the ability to frequently and reliably check for ketones if needed (88).

### 2.3 Combination of rtCGM/CSII

Some patients require and benefit from a combination of both CSII and rtCGM. When used in combination this is termed sensor augmented pump (SAP). In some SAP the rtCGM simply provides information to the wearer, allowing them to make manual adjustments to their CSII. More recent SAPs have low glucose suspend or predictive low glucose suspend (PLGS) features, which will pause insulin delivery when glucose levels begin to fall below a certain level. The most recent advance in SAP is the use of a hybrid closed loop system, which can automatically make adjustments to the wearer’s basal insulin in the presence of both hypo- and hyperglycaemia.

A definition of the different types of SAP can be found in **Box 2**.

Box 2 **Definitions of SAP (73).**

1^st^ generation SAP - the user has to make basal rate adjustments manually.

2^nd^ generation SAP – “the insulin dosing software and the rtCGM values are coupled which allows for automated suspension of basal insulin delivery in response to a predicted or detected low glucose level”.

Hybrid closed loop – maintain glucose levels within a target range through the use of a computerized algorithm to adjust the basal rate of insulin and administer corrective bolus doses (74).

Sensor augmented and closed loop technology have been studied in pregnancy since the early 2000’s and have shown some benefits in selected groups of patients.

Sensor augmented pump therapy with low glucose suspend has been shown to reduce HbA1c levels without increasing hypoglycaemia compared to standard treatment in observational studies (91). When compared to CSII without rtCGM, individuals using senor augmented pumps with low glucose suspend features had better third trimester glycaemic control, although no other differences, namely in macrosomia or pre-term birth were noted (92).

Reassuringly, these changes do not correlate with any increased risk in diabetic ketoacidosis (DKA) (93).

The initial studies of closed loop insulin delivery (where basal insulin levels are automatically adjusted to bring glucose levels within a target range) were studied in small numbers of women with type 1 diabetes over very short periods of time. These early studies demonstrated the safety of closed loop systems for short term use and paved the way for larger studies which demonstrated improved safety and less nocturnal hypoglycaemia (94, 95). In more advanced closed loop studies, TIR approached 69% in studies which evaluated pregnancy, labour and delivery. TIR approached 80% during labour itself and closed loop technology offered less hypoglycaemia than standard sensor augmented pump (96–98).

Due to the tight glycaemic control required in pregnancy, currently only one commercially available closed loop system is approved for use in pregnancy – CamsAPS FX (99). This closed loop system allows the user to personalise their glucose target, allowing them to achieve tighter control. Other commercially available closed loop systems do not allow the patient to reduce the target glucose to the levels required in pregnancy, but are often used either off-label or as SAP during pregnancy (100, 101). There are a number of ongoing randomised controlled trials aiming to evaluate commercial closed loop technology in pregnancy (102), NCT03774186, NCT04902378, NCT04520971, NCT04938557.

From a patient-reported outcome perspective, both benefits and burdens of closed-loop systems were described. Women reported having a sense of peace of mind and trust in the ease of use, however others described frustration with technical issues and being attached to diabetes related devices on a constant basis (103). While these considerations are very important they are not unique to pregnancy and commonly affect non-pregnant adults with type 1 diabetes (104).

### 2.4 Other

#### 2.4.1 Telemedicine

Telemedicine or telehealth is defined as “technology-based virtual platform to deliver various aspects of health information, prevention, monitoring, and medical care” (105). It was already increasingly used prior to the Sars-CoV-2 pandemic however its use soared during this time due to universal lockdown restrictions (106). Telemedicine offers increased convenience and flexibility and is generally acceptable to patients however to be beneficial in PGDM hard outcomes like glycaemic control and fetal outcomes need to be evaluated (107). A systematic review of the use of telemedicine in treating both PGDM and gestational diabetes (GDM) did not find any benefit in maternal or fetal outcomes, although very few studies evaluated women with PGDM (108). In studies that did include small numbers of patients with PGDM, telemedicine resulted in fewer GP and nurse visits (109), improved satisfaction and quality of life (110) and some slight improvements in glycaemic control (111).

Further evaluation is needed before telemedicine can become a routine part of care for pregnant women with PGDM.

#### 2.4.2 Smart pens

Smart pens are reusable insulin delivery devices which can help patients track their timing and doses of insulin. This prevents inadvertent insulin delivery when patients forget about previously administered doses and improves glycaemic control by helping to reduce the frequency of missed doses. Such interventions have been shown to improve HbA1c levels and are cost-effective in non-pregnant population (112). They are increasingly used and are highly acceptable to patients and improve confidence in diabetes self-management in both type 1 and type 2 diabetes (113, 114). Although it is reasonable to assume that these benefits would translate to women with PGDM there is no evidence in this area.

#### 2.4.3 Bolus calculating apps

Another innovation which has shown promise in the management of type 1 diabetes is the use of applications which facilitate carbohydrate counting and bolus calculation. A number of applications are available to facilitate carbohydrate counting, however more recent developments include the launch of food identification software that calculates the carbohydrate content of food using photographs (115, 116). This software can improve carbohydrate counting and HbA1c in a population of young adults, although its use is limited and it remains unvalidated in pregnancy (117).

## 3 Conclusion

In conclusion, a number of technological developments have improved the care for women with diabetes. Advancements in rtCGM and to a lesser extent isCGM offered greater convenience for patients, and have translated in improvements in clinical outcomes. CSII therapy has shown more conflicting results, however it has a number of benefits outside of pregnancy and the numbers of women entering pregnancy using CSII is likely to increase. Hybrid closed loop technology has shown significant promise in pregnancy. As this technology advances, becomes more widespread and cost-effective a greater number of women will be able to avail of its use - offering better glycaemic control and pregnancy outcomes in the future.

## Author contributions

CN performed the data search and reviewed articles; AE drafted the manuscript; CN and FD contributed to the original idea and design of the study. All authors contributed to the article and approved the submitted version.
